# Biosafety of rabies virus and related lyssaviruses: Risks, precautions, and knowledge gaps in diagnostic and research laboratories

**DOI:** 10.1371/journal.pntd.0014757

**Published:** 2026-10-05

**Authors:** Stuart D. Blacksell, Khanh Kim Le, Paola De Benedictis, Morgane Gourlaouen

**Affiliations:** 1 Mahidol Oxford Tropical Medicine Research Unit, Faculty of Tropical Medicine, Mahidol University, Bangkok, Thailand; 2 Centre for Tropical Medicine and Global Health, Nuffield Department of Medicine, University of Oxford, Oxford, United Kingdom; 3 Istituto Zooprofilattico Sperimentale delle Venezie, Food and Agriculture Organization/National Reference Centre for Rabies and World Organisation for Animal Health Reference Laboratory for Rabies, Legnaro, Padua, Italy; 4 Food and Agriculture Organization of the United Nations (FAO), Rome, Italy; CIRAD, ZIMBABWE

## Abstract

Lyssaviruses cause rabies in mammals, a viral encephalomyelitis that is almost always fatal once clinical symptoms develop and can be transmitted to humans. Given this high fatality rate, strict biosafety measures are essential during laboratory handling of potentially infectious materials. This review critically assesses current biosafety practices in rabies diagnostic and research laboratories by examining principal risk factors, transmission pathways, and the effectiveness of existing preventive measures. We conducted a comprehensive review of the literature on biosafety risks associated with handling rabies virus and related lyssaviruses, including the use of personal protective equipment, sample handling procedures, and chemical and physical methods for virus inactivation. Particular attention was given to documented cases of laboratory-acquired rabies and their implications for laboratory safety. Although laboratory-acquired infections remain rare, their occurrence highlights important gaps in current biosafety practices. The effectiveness of disinfection and inactivation methods varies widely, especially in resource-limited settings, and key diagnostic procedures, such as the direct fluorescent antibody test, require validation under biosafety constraints. Significant knowledge gaps remain regarding infectious dose, the effectiveness of decontamination methods, and diagnostic standardisation. Strengthening evidence-based biosafety protocols and validating inactivation methods are essential to ensure safer rabies diagnostics and to support effective rabies control, particularly in low-resource settings.

## Author summary

Rabies is a viral disease that is almost always fatal once symptoms develop and remains neglected in many parts of the world where laboratory resources are limited. Diagnostic and research work involving rabies virus and related lyssaviruses requires handling potentially infectious materials, placing laboratory staff at risk if appropriate biosafety measures are not in place. Although international guidance exists, many recommendations are based on limited evidence and may be difficult to apply in low-resource settings.

In this review, we examined the available evidence on biosafety risks associated with rabies and related lyssaviruses in diagnostic and research laboratories. We summarised what is known about transmission routes, laboratory-acquired infections, vaccination of staff, and the effectiveness of chemical and physical methods used to inactivate the virus. We also identified key knowledge gaps, particularly around disinfection practices, necropsy procedures, and access to pre-exposure vaccination.

Addressing these gaps is essential to protect laboratory personnel and strengthen rabies control efforts globally, especially in endemic and resource-limited settings.

## Introduction

Recent work has sought to strengthen the sustainable implementation of biological risk management in laboratories, particularly in low-resource settings. The Biosafety Research Roadmap project [1] seeks to enhance laboratory biological risk management by establishing an evidence base for biosafety interventions, including engineering controls, and by identifying practical, context-appropriate biosafety solutions for resource-limited environments. The project’s findings will help inform strategic decision-making regarding global health security and investments in laboratory systems. This initiative provides a comprehensive assessment of the existing evidence base for laboratory biological risk management, facilitating access to relevant research, identifying critical research and capability gaps, and providing recommendations for integrating evidence-based approaches into biosafety and biosecurity frameworks in low-resource settings.

The handling of rabies virus in laboratory settings predates the formal development of biosafety as a structured discipline. Early experimental work on rabies in the late 19th century by Louis Pasteur and Émile Roux involved serial passage of virus in animals and empirical approaches to virus attenuation and preservation, conducted without the benefit of modern containment infrastructure or formal risk assessment frameworks. Modern summaries of rabies history highlight how laboratory practices evolved alongside advances in virology, cell culture systems, and vaccine production technologies [2,3].

During the 20th century, the introduction of fixed rabies virus strains, laboratory-adapted variants with stable and predictable phenotypic characteristics used for research and vaccine production, led to increased manipulation of high-titre virus stocks. Recognition of laboratory-associated infections, including documented aerosol-related cases, progressively shaped the development of engineering controls, containment levels, and pre-exposure vaccination policies for laboratory personnel. The evolution of rabies research, therefore, mirrors the broader maturation of laboratory biosafety, transitioning from empirical handling of highly pathogenic virus to structured biological risk management grounded in containment principles, validated inactivation methods, and occupational health safeguards. This historical trajectory underscores that advances in rabies control have depended not only on scientific innovation but also on the parallel development of systematic biosafety practices.

This review specifically focuses on biosafety-relevant evidence and gaps associated with rabies and related lyssaviruses in diagnostic and research laboratory settings. Unlike previous reviews, this article synthesises biosafety evidence across diagnostics, necropsy, vaccination, and decontamination practices, with specific emphasis on operational risks and evidence gaps in low-resource laboratory settings [1,2,4,5].

## General characteristics

Lyssaviruses (genus *Lyssavirus*, family *Rhabdoviridae*) are highly neurotropic pathogens responsible for rabies, a fatal encephalomyelitis that affects mammals, including humans [2]. These viruses exhibit a characteristic bullet-shaped morphology, measuring approximately 180 nm in length and 75 nm in width. They have a viral envelope covered by a 10 nm peplomer glycoprotein spike layer, which plays a crucial role in host cell attachment and entry [6].

At the time of writing, the genus *Lyssavirus* currently comprises 18 species recognised by the International Committee on Taxonomy of Viruses (ICTV), together with additional tentative or unclassified lyssaviruses such as Phala bat lyssavirus (Table 1) [7]. Lyssaviruses are grouped into phylogroups based on antigenic and genetic similarity, with phylogroup I viruses generally neutralised by current vaccines, whereas phylogroups II and unassigned lyssaviruses may not be effectively neutralised. Several additional lyssavirus detections have been reported but not yet formally ratified as distinct species, reflecting ongoing taxonomic revision. RABV is the most widespread and the only species with a broad host range, enabling its persistence across diverse ecological and geographical environments [8,17]. In contrast, other lyssaviruses have more restricted host reservoirs, primarily infecting bat species with limited spillover events to other mammals [18–21]. Although rare, exposure to non-RABV lyssaviruses, especially those that are divergent, is worrisome because there are no efficacious biologicals to prevent infection.

**Table 1:** Recognised lyssavirus species and related unclassified viruses, phylogroup classification, reservoir associations, geographic distribution, biosafety classification, vaccine cross-protection, and documented human infections. Information has been synthesised from the current International Committee on Taxonomy of Viruses (ICTV) taxonomy together with guidance from the World Health Organization (WHO), the World Organisation for Animal Health (WOAH), *Biosafety in Microbiological and Biomedical Laboratories* (BMBL6), and peer-reviewed literature on lyssavirus ecology, epidemiology, vaccine cross-protection, and laboratory biosafety [2,4,7–15]. This table represents an original synthesis and is not reproduced from any single published source.

Lyssavirus species	Phylogroup	Putative Primary Reservoir^¶^	Documented Spillover Hosts	Risk Group^†^	BMBL6 BSL^*^	Geographic Distribution	Protection by Current Rabies Vaccines^#^	Documented Human Cases
Rabies virus (RABV)	I	Carnivores (particularly dogs and wildlife species) and multiple bat species	Multiple mammals including humans	2 or 3	2	Worldwide	Supported by available evidence	~59,000 estimated human deaths annually
Australian bat lyssavirus (ABLV)	I	Pteropodidae (flying foxes) and insectivorous bats (*Saccolaimus flaviventris*)	Humans	2 or 3	2	Australia	Supported by available evidence	4 fatal
Duvenhage virus (DUVV)	I	Insectivorous bats (genus *Nycteris*)	Humans	2 or 3	2	Africa	Supported by available evidence	3 fatal
European bat lyssavirus 1 (EBLV-1)	I	*Eptesicus serotinus* (Serotine bat)	Humans, terrestrial mammals	NC	2	Europe	Supported by available evidence	1 fatal
European bat lyssavirus 2 (EBLV-2)	I	*Myotis daubentonii* and *Myotis dasycneme*	Humans	NC	2	Europe	Supported by available evidence	2 fatal
Bokeloh bat lyssavirus (BBLV)	I	*Myotis nattereri*	ND	NC	2	Europe	Predicted from available evidence	None reported
Aravan virus (ARAV)	I	*Myotis blythii*	ND	NC	2	Central Asia	Predicted from available evidence	None reported
Khujand virus (KHUV)	I	*Myotis mystacinus*	ND	NC	2	Central Asia	Predicted from available evidence	None reported
Irkut virus (IRKV)	I	*Murina leucogaster*	Humans	NC	2	Russia, East Asia	Predicted from available evidence	1 fatal
Gannoruwa bat lyssavirus (GBLV)	I	*Pteropus medius* (tentative association)	ND	NC	NC	Sri Lanka	Insufficient evidence	None reported
Kotalahti bat lyssavirus (KBLV)	I	*Myotis brandtii* (detected)	ND	NC	NC	Europe	Supported by available evidence	None reported
Taiwan bat lyssavirus (TWBLV)	I	*Pipistrellus abramus*	ND	NC	NC	Taiwan	Predicted from available evidence	None reported
Lagos bat virus (LBV)	II	Fruit bats (family Pteropodidae, e.g., *Eidolon helvum*)	Domestic animals	NC	3	Africa	Not supported by available evidence	None confirmed
Mokola virus (MOKV)	II	Unknown (rodents suspected)	Domestic animals, humans	3	3	Africa	Not supported by available evidence	2 reported human cases (1 fatal, 1 survived)
Shimoni bat virus (SHIBV)	II	*Hipposideros commersoni*	ND	NC	3	Kenya	Not supported by available evidence	None reported
West Caucasian bat lyssavirus (WCBV)	Not assigned to phylogroup I or II	*Miniopterus schreibersii*	Domestic cat	NC	3	Europe	Not supported by available evidence	None confirmed
Ikoma lyssavirus (IKOV)	Not assigned to phylogroup I or II	Likely bats (reservoir uncertain)	African civet (spillover)	NC	3	Africa	Not supported by available evidence	None reported
Lleida bat lyssavirus (LLEBV)	Not assigned to phylogroup I or II	*Miniopterus schreibersii*	ND	NC	NC	Europe	Not supported by available evidence	None reported
Phala bat lyssavirus (PBLV)^‡^	Unclassified	*Nycticeinops schlieffeni*	ND	NC	NC	South Africa	Unknown; insufficient evidence	None reported

¶Putative primary reservoir associations are based on the best available ecological, epidemiological, virological, and molecular evidence and may change as additional surveillance and virus discovery studies become available.

†Details obtained from ABSA International Risk Group Database [16]. Where variation occurs, it is due to differences in national and international biosafety frameworks. For example, BMBL6 classifies rabies virus and several phylogroup I lyssaviruses as BSL-2 under specified circumstances because licensed vaccines and post-exposure prophylaxis are available, whereas many other national authorities classify lyssaviruses as RG3. Note the distinction between BSL and RG in this instance.

*BMBL6 (USA) biosafety level (BSL) recommendations [4].

#Assessment of vaccine protection is based on the best available experimental, antigenic, immunological, and challenge-study evidence together with current WHO and ACIP recommendations. For recently recognised or poorly characterised lyssaviruses, predicted protection is inferred from phylogenetic relationships, antigenic similarity, and available cross-neutralisation data rather than direct vaccine efficacy studies. These assessments should be regarded as provisional and may change as additional experimental and epidemiological evidence becomes available.

‡Phala bat lyssavirus is listed by ICTV as a related, unclassified virus. The virus name and abbreviation are not official ICTV designations [7].

Abbreviations: NC, not classified; ND, not documented.

### Global burden of rabies

Model-based estimates of dog-mediated rabies mortality range widely [22], from approximately 20,000 to 175,000 human deaths annually. Early global estimates suggested approximately 55,000 human deaths per year [23], with subsequent modelling studies producing a broadly similar figure of 59,000 estimated deaths annually [24,25]. More recent approaches have yielded substantially lower estimates of approximately 14,000 deaths per year [26]. Despite these variations, all models agree that rabies mortality is profoundly underreported, particularly in Asia and Africa [27].

### Clinical disease

The incubation period of rabies is highly variable, ranging from a few days to several years. Incubation length is influenced by factors such as the type and anatomical location of exposure. Still, it does not appear to differ between clinical forms of disease, although the underlying mechanisms remain poorly understood. Although the incubation period in human rabies is most commonly 20–90 days, onset may occur within days or be delayed for months to years [28]. This variability in incubation has important implications for occupational exposure attribution and laboratory risk assessment. Prolonged incubation periods have been documented, including cases in immigrants to the United States with disease onset occurring 11 months to 6 years after presumed exposure [29], a reported 3-year incubation in China [30], and a case in Australia likely acquired 4.5–6 years earlier in Asia [31]. The probability of developing rabies following untreated exposure varies by anatomical site, occurring in approximately 50%–80% of head bites, 15%–40% of hand or arm bites, 3%–10% of leg bites, and around 0.1% following minor wounds or scratches contaminated with saliva [32]. Localised pain, paraesthesia, or pruritus at or near the exposure site occurs in 30%–70% of patients and may reflect viral infection of peripheral sensory ganglia; in some cases, early neurological symptoms may arise at sites distant from the original exposure [32,33].

Clinical illness typically begins with a prodromal phase lasting up to 10 days, characterised by non-specific symptoms, including fever, headache, fatigue, anxiety, and, frequently, pain, tingling, or pruritus at or near the site of exposure, an early hallmark of rabies infection [34,35]. This phase is followed by rapid progression to overt neurological disease [34]. Two principal clinical forms are recognised. Encephalitic (furious) rabies, the more common presentation, is characterised by agitation, anxiety, confusion, hallucinations, and autonomic dysfunction, with hydrophobia and aerophobia representing distinctive but inconsistently present features [36]. Paralytic (dumb) rabies develops more insidiously and is characterised by ascending flaccid paralysis, often leading to misdiagnosis as poliomyelitis or botulism, in the absence of marked behavioural disturbances. Regardless of clinical presentation, rabies is almost invariably fatal once neurological signs develop [37].

### Diagnostics

Due to the lack of pathognomonic signs, international organisations agree on the centrality of laboratory confirmation in diagnosing human clinical rabies [38,39]. In this context, the viral agent has to be identified mainly *post-mortem* [5,9] from brain tissue collected through skull dissection or minimally invasive approaches via the occipital foramen or retro-orbital routes [5,40]. Brain samples are tested using the gold-standard diagnostic techniques, primarily the direct fluorescent antibody (DFA) test, and pan-lyssavirus molecular methods, which have more recently been included among the official standards for rabies diagnostic tools [5,9]. Rapid immunochromatographic tests (also known as Lateral Flow Devices, or LFDs) are increasingly used as screening tools for field surveillance, with potential biosafety advantages due to reduced handling of infectious brain tissue. However, LFD results are subject to laboratory confirmation using a gold-standard test [9,41,42]. While post-mortem diagnosis remains the most definitive way to confirm rabies, human diagnosis is frequently attempted before death. This includes collecting and testing saliva, performing a skin biopsy, and using molecular techniques and DFA, with DFA applied solely to the skin biopsy. In addition to saliva and skin biopsy specimens, cerebrospinal fluid (CSF) may be tested for rabies virus-neutralising antibodies using seroneutralisation assays such as Rapid Fluorescent Focus Inhibition Test (RFFIT) or the Fluorescent Antibody Virus Neutralisation (FAVN) test. This is particularly important in unvaccinated individuals, where the presence of neutralising antibodies in CSF supports a diagnosis of clinical rabies. Together, these diagnostic approaches differ substantially in biosafety risk, particularly with respect to tissue manipulation, aerosol generation, and containment requirements, underscoring the need for context-specific risk assessment.

### Pre- and post-exposure prophylaxis

Rabies remains an incurable disease [43], with only a few documented cases of survival. As a result, pre-exposure prophylaxis (PrEP) plays a crucial role in protecting populations at high risk of exposure, including laboratory personnel. Only WHO-prequalified rabies vaccines should be used for occupational pre-exposure and post-exposure prophylaxis to ensure quality, safety, and immunogenicity. Regardless of prior vaccination status, post-exposure prophylaxis (PEP) should be initiated promptly after exposure. PEP includes immediate and thorough wound washing, rabies vaccination, and the administration of rabies immune globulin or monoclonal antibodies, in the absence of PrEP and according to the exposure risk category [10,44–46]. Of note, individuals who have received pre-exposure prophylaxis still require post-exposure vaccination following exposure, but do not require passive immunisation and only need booster doses. In many low-resource settings, particularly in parts of sub-Saharan Africa, routine serological monitoring is not feasible due to limited laboratory capacity and cost constraints. In such contexts, reliance on scheduled booster doses rather than titre-guided monitoring may be a pragmatic alternative.

### Modes of transmission

#### Bite transmission.

Bite exposure is the principal route of transmission for rabies virus and other lyssaviruses, occurring through the introduction of virus-laden saliva into tissues through breaches in the skin [2,36]. Transmission may also occur through contamination of mucous membranes or pre-existing wounds, although these routes are less common. The risk of infection varies with factors such as bite location and severity; highly innervated areas (e.g., head and hands) are associated with a higher risk and shorter incubation periods [32]. Because infectiousness cannot be reliably determined in live animals, all bites from suspect animals should be considered potentially infectious [2,5].

#### Transplantation and blood exposure.

Human rabies transmission through transplantation, while rare, has been documented repeatedly across corneal and solid organ grafts over several decades. Between 1978 and 1994, eight fatal cases were linked to corneal transplantation in the United States, Europe, and Asia, with death occurring within weeks to months after surgery [47,48]. Donor-derived rabies from solid organ transplantation was first clearly recognised in 2004 in the United States, where a single donor infected four recipients, followed by similar fatal clusters in Germany in 2005. Since then, sporadic cases and small outbreaks have been reported worldwide, including in the United States, Kuwait, Saudi Arabia, China, and Syria, involving kidney, liver, lung, heart, and vascular grafts [48–52]. Incubation periods have ranged from several weeks to over a year, and all reported cases were fatal, underscoring the persistent risk of rabies transmission through transplantation and the critical importance of donor exposure assessment and surveillance [48]. Beyond transplantation, other non-bite transmission routes have been documented, including exposure to infected blood [53,54].

#### Vertical transmission.

Although several reports have explored the possibility of vertical (mother-to-child) transmission in both animals and humans, documented neonatal rabies attributable to confirmed transplacental transmission remains exceedingly rare. Interpretation is complicated by potential postnatal exposure, and the epidemiological evidence remains inconclusive [55].

#### Bat-associated rabies and cryptic exposures.

In the American continent, most recent indigenous human rabies cases have been associated with bat-associated rabies virus variants rather than dog-mediated transmission [2,3,48,56,57]. Bat-associated rabies presents distinct epidemiological and clinical challenges. Unlike dog bites, bat exposures are often minor, superficial, or unrecognised, and several human cases have occurred without a clear history of a bite [3,57]. Small puncture wounds may go unnoticed, particularly during sleep or in vulnerable individuals.

Public health authorities in North America, Europe, and Australia recognise the risk posed by cryptic bat exposures. Current guidance recommends post-exposure prophylaxis when direct contact with a bat cannot be confidently excluded, including scenarios involving unattended sleeping persons, young children, individuals with cognitive impairment, or persons under the influence of alcohol or sedatives in enclosed spaces where a bat has been present [10,58,59]. These recommendations reflect the difficulty of reliably excluding minor percutaneous exposure.

In addition to RABV circulating in American bats, several phylogroup I lyssaviruses maintained in bats, including Australian bat lyssavirus, Duvenhage virus, and European bat lyssaviruses 1 and 2, have been shown to cause fatal human infections [2,11,21,31,60]. Although current rabies vaccines are considered protective against phylogroup I bat lyssaviruses, they are unlikely to provide protection against more divergent lyssaviruses in phylogroup II [11,12,61]. This distinction has important implications for laboratory biosafety and occupational risk assessment when handling bat-derived specimens.

From a laboratory perspective, bat rabies introduces additional biosafety considerations. Field collection of bat specimens may involve wildlife handlers and surveillance personnel operating outside standard laboratory infrastructure. These individuals may not have the same level of biosafety training or vaccination coverage as laboratory staff. Moreover, the small size of bats increases the likelihood of unnoticed exposure during handling [57]. Accordingly, risk assessment and pre-exposure vaccination policies should explicitly include field personnel engaged in bat surveillance activities. Public health responses to bat incursions in residential or institutional settings typically involve risk assessment, capture and testing of the bat where possible, and administration of PEP when exposure cannot be confidently excluded.

#### Laboratory-acquired infections.

Only two laboratory-acquired rabies virus infections have been documented (Table 2), which is likely attributable in part to the widespread use of pre-exposure vaccination among laboratory personnel. The first reported case was in 1972, involving a 56-year-old veterinarian working in a rabies vaccine laboratory who likely became infected through aerosol inhalation during virus cultivation and electron microscopy [62]. The second case was recorded in 1977 in New York, involving a 32-year-old laboratory worker who was thought to have contracted rabies after inhaling aerosols from a leaking pharmaceutical manufacturing machine. Although he had previously worked with the same device and probably been exposed to RABV before, this specific exposure was considered the likely cause of his infection [63]. Both confirmed cases of laboratory-acquired rabies happened in industrial or laboratory environments involving aerosol-generating procedures. They were not linked to animal bites or direct inoculation. These instances support existing biosafety guidelines that stress aerosol containment, engineering controls, and pre-exposure vaccination for personnel handling RABV.

**Table 2:** Published laboratory-acquired rabies virus infections relevant to biosafety risk assessment. Documented laboratory-associated rabies virus infections in which occupational exposure was considered the most likely source of infection. Information was synthesised from the original case reports and biosafety literature and is presented as an original compilation, not reproduced from any single source.

Case	Year	Country/Location	Age/Sex	Occupation/Setting	Exposure circumstances	Virus strain (reported)	Outcome	Reference
Laboratory-acquired rabies (aerosol exposure)	1972 (reported 1973)	United States	56-year-old male	Veterinarian working in a laboratory manufacturing rabies vaccine	Presumed inhalation of aerosolised rabies virus generated during vaccine production and laboratory manipulation. No history of animal bite or recognised percutaneous exposure was identified.	Fixed rabies virus strain	Fatal rabies	[62]
Laboratory-acquired rabies (manufacturing equipment failure)	1977	United States (New York)	32-year-old male	Laboratory worker in pharmaceutical manufacturing facility	Presumed inhalation of aerosolised rabies virus following leakage from pharmaceutical manufacturing equipment. Although previous occupational exposure could not be excluded, the documented equipment failure was considered the most likely source of infection.	Not specified	Fatal rabies	[63]

### Infectious dose

The viral infectious dose required to cause clinical disease varies depending on virus species, strain, host, and route of exposure [43]. For RABV, despite being a multi-host pathogen with a remarkable ability to cross species barriers, the capability to infect new hosts is far from uniform, as different RABV lineages and strains exhibit variable competence in infecting and transmitting within diverse mammalian species, as illustrated by the higher virulence of canine RABV in foxes compared to that of fox RABV in domestic dogs, suggesting a strong unidirectional host evolutionary effect [18,64]. Several studies have demonstrated that RABV can establish clinical disease following exposure to minimal viral particles when introduced via peripheral routes, such as intramuscular or subcutaneous injection, though higher doses are associated with shorter incubation periods and higher fatality rates [65,66]. Viral shedding through saliva from infected animals displaying clinical signs also varies extensively, especially among viral species, with RABV generally shedding more than non-RABV lyssaviruses. Experimental and field studies suggest that viral shedding may be intermittent during early clinical stages, complicating exposure risk assessment. These observations remind us that host switching requires considerable adaptive effort, leading to host-specific and independent RABV cycles and limited spillover events of non-RABV lyssaviruses. However, given the variety of circumstances, any potential exposure should not be overlooked, and the precise infectious dose required for viral transmission via different modes remains largely undetermined [43]. As with RABV, diverse pathogenicity has been observed among members of the genus. For example, Duvenhage virus (DUVV) and Lleida bat lyssavirus (LLEBV) have shown little or no neuroinvasiveness following intramuscular injection, despite being pathogenic when injected intracerebrally in rodents [18,67,68].

## Biosafety knowledge and requirements

### Risk group classification

Lyssaviruses are generally categorised as risk group (RG) 3 pathogens for humans and animals [4,43,69–72]. Risk group classification reflects the inherent hazard of an infectious agent, including disease severity and the availability of effective prophylaxis or treatment. In contrast, biosafety level (BSL) defines the laboratory biocontainment practices, safety equipment, and procedural controls required for its safe handling. Although *Biosafety in Microbiological and Biomedical Laboratories, 6th edition* (BMBL6) [4], specifies RABV and several phylogroup I lyssaviruses to be worked with at BSL-2 because effective prophylaxis is available (see Tables 3 and 4), many other national and international frameworks classify lyssaviruses as RG3 without specifying the BSL. Regardless of risk group designation, many laboratory activities involving live virus require BSL-3 containment because of disease severity and aerosol exposure risks. Laboratory and animal-handling activities involving RABV can be further stratified by exposure risk (See Table 5). Low-risk activities include work with fixed or properly inactivated specimens, serological assays, and molecular testing of validated non-infectious material, as infectious aerosols have not been demonstrated to pose a hazard during routine diagnostic procedures [4]. Although some serological and neutralisation assays involve concentrated viral antigens, these materials are validated as inactivated prior to use and do not represent an aerosol transmission risk when handled appropriately. The documented laboratory-acquired cases occurred in industrial or high-titre virus propagation settings rather than during routine diagnostic workflows.

Moderate-risk activities include handling fresh clinical specimens outside the central nervous system, such as saliva or skin biopsies; controlled handling of potentially infected animals, and manipulation of fixed or attenuated rabies virus strains, where exposure may occur through sharps injuries or contact with mucous membranes or non-intact skin [4]. Because rabies cannot be definitively confirmed in live animals prior to euthanasia and laboratory testing, all suspect animals should be treated as potentially infectious for the purposes of risk assessment. High-risk activities include necropsy or autopsy procedures involving the brain or spinal cord, opening the skull, or removing central nervous system (CNS) tissue outside a biological safety cabinet, handling live rabies virus or infected animals, and procedures with a high likelihood of sharps injuries, animal bites, or splashes. These activities involve central nervous system tissues, which are consistently associated with high viral loads during clinical rabies [34] and have been associated with laboratory-acquired infections, necessitating control measures, including enhanced personal protective equipment and rigorous decontamination. Handling of inactivated specimens and molecular diagnostics using validated inactivation methods may be conducted under BSL-2 conditions. In contrast, the propagation of live rabies virus, the manipulation of concentrated virus stocks, or the necropsy of CNS tissue outside containment requires BSL-3 containment (See Table 5 for full details).

### Risk mitigation strategies

#### Persistence of RABV in carcasses.

Understanding the persistence of RABV in decomposing carcasses is crucial for accurate post-mortem diagnosis, especially in forensic and epidemiological investigations [5,9]. Generally, RABV RNA or antigen is detectable for extended periods [73–76]. Studies have demonstrated that DFA-positive RABV antigens remain detectable for up to 36 days at 4 °C [76] and that, using hemi-nested RT-PCR (HnRT-PCR), viral RNA could be detected for up to 70 days at 4 °C and 48 days at both 25 °C and 35 °C [76]. Other studies confirmed that RABV RNA could be detected longer than viral antigen in brains collected from decomposed carcasses [74], with detection lasting up to 76 days in buried/exhumed dog carcasses [75]. However, DFA and PCR RABV positivity do not necessarily indicate virus viability or infectious risk. Virus viability may be demonstrated using mouse intracerebral inoculation tests (MI) or rabies tissue culture infection tests (RTCIT). Generally, RABV remained infectious for much shorter periods in decomposed specimens, although this is temperature-dependent, with viability extending to longer periods at lower temperatures. This has been demonstrated in decomposed samples, with viability observed at 4 °C for 18 days, but only for 3 days at 25 °C and 35 °C [76]. Similarly, RABV-infected canine brains stored at room temperature (25–29 °C) were viable for only 48 h using the MI test [73]. Field investigations have yielded discordant infectivity and diagnostic results from decomposed brain samples, as described by David and colleagues, who isolated RABV via the MI test from three of ten DFA-negative but RABV RT-PCR-positive samples [74]. These findings demonstrate that while RABV RNA or antigen can be detected in decomposed tissue for extended periods, the associated risk of infection is relatively low, though not quantifiable, when handling potentially RABV-contaminated materials. Carcasses, tissues, disposable test materials and other waste from rabies-suspect animals should be treated as potentially infectious and managed under a documented, risk-based waste-management procedure. Waste should be segregated, contained and decontaminated as close as practicable to the point of generation. Validated autoclaving or incineration should be used where available, followed by disposal in accordance with national requirements. Alternative carcass-disposal methods should be selected only after a local risk assessment and approval by the relevant veterinary, public health and environmental authorities [9,13,42].

#### Recommendations to prevent laboratory- or occupationally-acquired rabies infection.

Aerosol generation during manipulation can be minimised by performing procedures within a certified Class II biological safety cabinet, using sealed centrifuge rotors, avoiding vortexing or high-pressure washing outside containment, and implementing engineering controls appropriate to the procedure [4,5]. According to guidance from the WHO [5] and the US Centers for Disease Control and Prevention/Advisory Committee on Immunization Practices (CDC/ACIP) [58,59], laboratory staff working with rabies virus or related specimens are considered high-risk and should receive pre-exposure prophylaxis (PrEP). Laboratory personnel handling live rabies virus, infected animals, or diagnostic specimens are considered occupationally at increased risk and should receive PrEP according to national recommendations. PrEP consists of a primary 2-dose rabies vaccination schedule administered intramuscularly or intradermally, with doses given on Days 0 and 7 (see Tables 3 and 4). Only WHO-prequalified rabies vaccines should be used for occupational pre-exposure and post-exposure prophylaxis to ensure product quality, safety, and immunogenicity in both endemic and non-endemic settings. Although WHO recommends a 2-dose primary series (days 0 and 7), some national programmes continue to administer a third intramuscular dose, and local guidelines should be followed. For individuals with ongoing occupational exposure, WHO and CDC/ACIP recommend periodic serological monitoring to ensure adequate rabies virus-neutralising antibody levels, with a protective threshold of ≥0.5 IU/mL and booster vaccination if titres fall below this level; Australian guidance also recommends serological monitoring and booster doses for those with ongoing or occupational exposure risk, without specifying a fixed testing interval. Very limited data are available to define minimum protective antibody thresholds for non-rabies lyssaviruses. The immune status of laboratory staff should also be considered, particularly in individuals with immunocompromising conditions, as this may increase the risk of PrEP or PEP failure [78].

**Table 3:** Comparison of selected World Health Organization (WHO) [10], United States Centers for Disease Control and Prevention/Advisory Committee on Immunization Practices (CDC/ACIP) [58], and Australian [77] recommendations for rabies pre-exposure prophylaxis (PrEP) and post-exposure prophylaxis (PEP). Recommendations have been summarised and harmonised from the respective national and international guidelines and are presented as an original compilation rather than reproduced from any single source.

Context	WHO	CDC/ACIP	Australia
PrEP—Who should receive	High-risk groups including veterinarians, bat handlers, wildlife workers, and travellers to endemic areas with limited access to PEP	People in ACIP Risk Categories 1–5, according to the nature and duration of risk, including veterinarians, animal handlers, and travellers to endemic areas	Increased-risk groups including veterinarians, bat handlers, wildlife workers, laboratory personnel working with lyssaviruses, and travellers to endemic areas with limited access to PEP
PrEP—Vaccine and schedule	Days 0 and 7: IM or 2-site ID	HDCV or PCECV on Days 0 and 7, IM	Days 0 and 7 (IM or 2-site ID), or Days 0, 7 and 21–28 (IM or ID)
PrEP—Monitoring/boosters	Periodic serology and booster vaccination for people at continuing high risk; generally not required for most travellers	Category 1: titre every 6 months; Category 2: every 2 years; Category 3: one-time titre during Years 1–3 or one booster between Day 21 and Year 3; Categories 4 & 5: no routine titre or booster	Serological monitoring and boosters according to ongoing occupational risk; after a 2-visit schedule, give one IM booster at 1 year if longer-term protection is required
PEP—Wound care	Immediate washing and flushing with soap and water for approximately 15 min; apply a virucidal agent where available	Immediate and thorough cleansing of all wounds with soap and water; use a virucidal agent where available	Immediate washing with soap and water for at least 15 min; apply a virucidal agent where available.
PEP—Vaccine unvaccinated	Two-site ID vaccine on Days 0, 3 and 7, or an approved IM regimen	HDCV or PCECV IM on Days 0, 3, 7 and 14; add Day 28 for immunocompromised people	IM vaccine on Days 0, 3, 7 and 14; add Day 28 and serology for immunocompromised people
PEP—Previously vaccinated	Two vaccine doses on Days 0 and 3, IM or ID; no RIG	HDCV or PCECV on Days 0 and 3, IM; no HRIG	Two vaccine doses on Days 0 and 3; no HRIG for previously vaccinated immunocompetent people.
PEP—RIG	Human or equine RIG for Category III exposures in previously unvaccinated people; infiltrate into and around wounds.	HRIG, 20 IU/kg, for previously unvaccinated people; infiltrate into and around wounds where possible and administer any remainder IM at a site distant from vaccine administration.	HRIG where indicated; administer as soon as possible and within 7 days of the first vaccine dose.
PEP—Key point	RIG is not indicated after a recognised completed PrEP or previous PEP course	Same as WHO	HRIG is not routinely indicated after recognised completed PrEP or previous PEP; exceptions may apply in severe immunocompromise.

Abbreviations: ACIP, Advisory Committee on Immunization Practices; HDCV, human diploid cell vaccine; PCECV, purified chick embryo cell vaccine; ID, intradermal; IM, intramuscular; RIG, rabies immunoglobulin (HRIG, human rabies immunoglobulin; ERIG, equine rabies immunoglobulin); RVNA, rabies virus-neutralising antibody.

Notes: Recommended IM vaccine dose: WHO, according to product specification; CDC, 1.0 mL; Australia, 0.5 or 1.0 mL according to the vaccine product. Recommended ID dose: 0.1 mL per site where applicable. Protective RVNA concentration: ≥0.5 IU/mL. RIG is administered once at the initiation of PEP, and the recommended dose should not be exceeded. ACIP Risk Category 1: rabies virus research and vaccine-production personnel; Category 2: rabies diagnostic laboratory personnel, bat handlers and spelunkers; Category 3: people with sustained risk of recognised exposure for more than 3 years; Category 4: people with risk of recognised exposure lasting no more than 3 years; Category 5: general population with no recognised risk factors for rabies exposure.

**Table 4:** Internationally adapted rabies pre-exposure prophylaxis (PrEP) risk categories and recommendations. Risk categories and recommendations for PrEP, rabies virus-neutralising antibody (RVNA) titre monitoring, and booster vaccination are adapted from the 2022 United States Advisory Committee on Immunization Practices (ACIP) rabies PrEP recommendations and the 2025 CDC guidance [58]. Occupational and exposure descriptions have been simplified for international application while preserving the original risk stratification.

Risk Category	Exposure Risk Description	Who fits here?	PrEP recommendation	Titre checks/Boosters
1	Highest risk	Rabies virus workers in laboratories (handling live virus)	Yes	Titre check every 6 months; booster if <0.5 IU/mL
2	Frequent risk	People frequently handling bats, wildlife, or diagnostic specimens (excluding propagation of live virus laboratories)	Yes	Titre check every 2 years; booster if <0.5 IU/mL
3	Infrequent risk but with risk of recognised exposure	Veterinarians, veterinary technicians, animal control, travellers to endemic areas with access to care	Yes	One-time titre check during Years 1–3 or one booster between Day 21 and Year 3 following the first 2-dose vaccination
4	Elevated risk, time-limited (≤3 years after PrEP)	Same population as risk category 3, but at a higher risk for ≤ 3 years after they receive PrEP	Yes	None
5	Lowest risk	General population with no recognised risk factors for rabies exposure	No routine PrEP	None

Abbreviations: ACIP, Advisory Committee on Immunization Practices; PrEP, pre-exposure prophylaxis; RVNA, rabies virus-neutralising antibody; IU, international units.

Monitoring post-vaccination immunity in staff should include assessment of rabies virus-neutralising antibodies using one of the internationally recommended reference tests (e.g., RFFIT or FAVN). In addition, commercially available assays such as the Bio-Rad Platelia Rabies II ELISA may serve as an adequate screening method to indicate whether antibody levels are consistent with protective immunity, provided that results are interpreted in light of the validated reference methods.

#### Management of laboratory incidents and accidents, and post-exposure prophylaxis.

Effective management of laboratory incidents and accidents is crucial for ensuring the safety of personnel handling infectious agents, such as lyssaviruses. PEP, as recommended by WHO [10] and CDC/ACIP [58], is essential for laboratory personnel exposed to lyssaviruses (see Table 3 for WHO/CDC/ACIP PEP guidance). This is especially the case for staff who are exposed to the highest-risk situations, including necropsy of infected CNS tissue and laboratory propagation of live virus stocks, which carry a risk of high-level exposure in the event of an accident or incident. In the event of accidental exposure, immediate wound care is crucial. Thoroughly washing the affected area with soap and water significantly reduces the risk of infection, followed by vaccination or vaccination booster and rabies immunoglobulin (RIG), depending on the vaccination status of the individual staff member (See Table 3).

**Table 5:** Recommended biosafety practices for processing potentially rabies virus-contaminated clinical specimens in diagnostic laboratories. The recommendations have been synthesised from guidance published by the WHO [5,13], WOAH [9], and BMBL6 [4]. The table represents an original synthesis and is not reproduced from any single published source.

Assay/Activity	Processing step	Prevention measures
Brain sample collection	Not applicable	Wear a cut-resistant metal mesh glove that reaches the forearm, a face shield (visor) or chemical splash goggles, an N95 or FFP2 respirator, a cap, protective coveralls, and two pairs of disposable gloves.
Brain sample handling (preparation, dilution)	Not applicable	Wear appropriate personal protective equipment (PPE), including gowns, and dedicated laboratory footwear (or disposable shoe covers) and two pairs of disposable gloves. Perform the operations in a Class II biological safety cabinet (BSC).
Direct fluorescent antibody	Preparation of smears or tissue impressions, acetone fixation and drying	Wear appropriate PPE, including gowns, disposable sleeves and dedicated laboratory footwear (or disposable shoe covers) and two pairs of disposable gloves. Perform the operations in a Class II BSC equipped with appropriate exhaust filtration for acetone vapours (if applicable).
	Reading and interpretation of the slide	Wear appropriate PPE, including gowns, and dedicated laboratory footwear (or disposable shoe covers) and disposable gloves.
Molecular tests	Nucleic acids extraction	Wear appropriate PPE, including gowns, disposable sleeves and dedicated laboratory footwear (or disposable shoe covers) and two pairs of disposable gloves. Perform the operations in a Class II BSC.
	Master mix preparation and nucleic acid addition	From this stage onwards, specimens may be considered non-infectious provided validated extraction and inactivation procedures have been completed.
Virus isolation attempt on cell cultures	Cell fixation and staining	Wear appropriate PPE, including gowns, disposable sleeves and dedicated laboratory footwear (or disposable shoe covers) and two pairs of disposable gloves. Perform the operations in a Class II BSC equipped with appropriate exhaust filtration for acetone vapours (if applicable).
	Reading and interpretation of the cell substrate	Wear appropriate PPE, including gowns, and dedicated laboratory footwear (or disposable shoe covers) and disposable gloves.
Abbreviations: BSC, Biological Safety Cabinet; PPE, Personal Protective Equipment.		

### Disinfection and decontamination

#### Chemical inactivation.

For clarity, chemical fixation and virus inactivation refer to processes applied to render specimens non-infectious for downstream analysis, whereas disinfection refers to the decontamination of surfaces, instruments, or environments. Various chemical agents can inactivate RABV at different exposure times and with varying levels of efficacy (Table 6). Evidence for RABV inactivation is extensive but highly context-dependent, with important implications for laboratory and necropsy biosafety. Studies have shown that an ascorbic acid (0.5 mg/mL) and copper sulfate (5 μg/mL) solution can eliminate the virus within 72 h [79], whereas hydrogen peroxide (3%) requires 2 h for inactivation [80]. More rapid inactivation has been observed with Virkon S (1%), which produced a >4-log_10_ reduction within 1 min in the presence of 10% foetal calf serum [81], whereas longer contact times (up to 1 h) may be required for heavily contaminated necropsy instruments under high organic load [84]. Isopropyl alcohol (70%) reduces the virus titres by >3 log within 20 s [81]. Paraformaldehyde (3% or 4%) has completely inactivated viral titres ranging from 10^2.33^ to 10^3^ fluorescent focus units (ffu)/mL within 30 min [81]. According to the World Organisation for Animal Health (WOAH), RABV can be inactivated using 10% formalin in phosphate-buffered saline (PBS). However, modifications to inactivation conditions may be necessary depending on the assay used [85]. Considering laboratory handling, it is worth mentioning that acetone fixation does not reliably inactivate RABV [86], raising concerns about its use in routine laboratory protocols, particularly for tissue preservation in DFA [84]. However, acetone has been demonstrated to inactivate RABV under specific conditions (37 °C for 4 h or 50 °C for ≥30 min) [82], though these methods are impractical due to time and safety [86]. Similarly, there is growing evidence that commonly used lysis buffers often fail to fully inactivate viral particles, particularly when applied without prior heat treatment or under high viral load conditions [87,88], raising concerns about the appropriateness of lowering biosafety containment levels during molecular biology procedures prior to extracting viral nucleic acids. The absence of evidence demonstrating complete lyssavirus inactivation by lysis buffers should not be interpreted as evidence that no residual risk exists.

**Table 6:** Experimental evidence for chemical, physical and thermal inactivation of rabies virus. Reported conditions and outcomes are summarised from published experimental studies. Results apply only to the virus strains, matrices, concentrations, temperatures and contact times evaluated and should not be interpreted as universally validated laboratory decontamination procedures.

Method	Experimental conditions and reported effect	Reference
Ascorbic acid and copper sulfate	Ascorbic acid 0.5 mg/mL plus copper sulfate 5 μg/mL completely inactivated fixed rabies virus after 72 h at 4 °C while retaining antigenicity	[79]
Hydrogen peroxide	Hydrogen peroxide 3% inactivated a Vero-cell-adapted fixed strain within 2 h	[80]
Virkon S	Virkon S 1% produced >4-log_10_ reduction within 1 min in medium containing 10% foetal calf serum	[81]
Isopropanol	Isopropanol 70% produced >3-log_10_ reduction within 20 s under the tested conditions	[81]
Ethanol	Ethanol 70% was ineffective under the tested experimental conditions	[81]
Paraformaldehyde	Paraformaldehyde 3%–4% for 30 min inactivated virus in infected cell cultures	[81]
Heated acetone fixation	Acetone fixation at 37 °C for 4 h or 50 °C for ≥30 min inactivated virus in tissue-impression smears	[82]
β-propiolactone	β-Propiolactone 0.1% at 37 °C for 2 h inactivated virus in tissue-impression smears	[82]
Heat	Heating at 56 °C for at least 30 min rendered high-titre mouse-brain suspensions non-infectious	[82]
Gamma irradiation	Approximately 1.2–1.6 megarads inactivated infected brain material; ≥1.6 megarads was required for CVS-11 suspensions	[83]

For vaccine production and research, β-propiolactone (BPL) and binary ethyleneimine (BEI) are widely used [89], as they effectively inactivate RABV while preserving antigenicity; however, their application in routine diagnostics requires further investigation [82]. Some traditional disinfectants used for necropsy tool decontamination, such as biguanides and quaternary ammonium compounds (QACs), do not reliably degrade RABV RNA, particularly under conditions of heavy organic contamination and when used at manufacturer-recommended concentrations [84]. Quaternary ammonium compounds are generally effective against enveloped viruses, including rabies virus, on clean environmental surfaces with minimal organic contamination. However, their activity is substantially reduced in the presence of blood, tissue, or other organic material. Consequently, QACs are most appropriate for routine environmental surface decontamination following thorough cleaning, whereas oxidising agents or phenolic disinfectants are preferred for heavily contaminated laboratory equipment and necropsy instruments. From a practical biosafety perspective, oxidising disinfectants (e.g., sodium hypochlorite and Virkon S) demonstrate the greatest overall efficacy for rabies virus decontamination, particularly under high-risk laboratory conditions. Aldehydes (e.g., formaldehyde and paraformaldehyde) provide highly effective virus inactivation for specimen fixation but present significant chemical hazards. Alcohols provide rapid inactivation on clean surfaces but have limited efficacy in the presence of organic material. Phenolic compounds are effective for instrument decontamination under defined conditions, whereas quaternary ammonium compounds are best reserved for cleaned environmental surfaces with low organic contamination [90–92]. However, both oxidising agents (i.e., 1% Virkon S for 1 h when decontaminating heavily contaminated necropsy instruments under high organic load, and 3% sodium hypochlorite for 30 min) and phenolic compounds (i.e., use dilution 5% or final concentration 10,950 ppm for 30–60 min) can effectively inactivate RABV on necropsy tools when used at higher-than-label concentrations and longer contact times, but their ability to degrade viral RNA declines after a few days, so freshly prepared solutions are recommended for reliable decontamination [84]. It should also be noted that, in the presence of organic contamination, common disinfectants may not be effective against viable RABV or RNA [84].

These findings highlight the need for rigorous, context-specific validation of inactivation protocols to ensure biosafety in laboratory and necropsy settings while maintaining the integrity of viral components for research and diagnostic applications. Studies on preservation and transport media show that FTA cards [93] and PrimeStore MTM [94] effectively inactivate the rabies virus while stabilising viral RNA for downstream molecular analysis. These materials render samples non-infectious at the point of collection or processing, thereby improving biosafety and enabling reliable diagnostic testing without compromising RNA integrity. However, conflicting evidence exists: viable virus has been detected when RABV LFD is used as a transport medium for PCR applications after treatment with extraction buffers [95]. In the conventional hierarchy of resistance to chemical disinfection, lipid-enveloped viruses such as RABV are generally more susceptible than non-enveloped viruses, mycobacteria and bacterial spores [90–92]. This relative susceptibility should not be interpreted as an absence of risk in infected tissues. Infectious RABV has been recovered from decomposing material for up to 18 days at 4 °C, compared with approximately 3 days at 25 °C or 35 °C [76]. Infected canine brain tissue retained infectivity for approximately 48 h at 25–29 °C [73]. By comparison, environmentally resistant organisms can remain viable substantially longer: *Bacillus anthracis* spores have been recovered from soil at animal carcass sites 3.8–10.4 years after host death [96]. Similarly, experimental studies have recovered viable *Bacillus* spores from indoor surfaces for up to 1,038 days [97]. Thus, RABV does not exhibit exceptional environmental persistence; however, low temperatures, protection within infected tissues and heavy organic contamination can prolong viability and reduce disinfectant efficacy. The principal biosafety concern is therefore the severe consequence of accidental exposure, combined with uncertainty about virus survival under specific handling and environmental conditions.

#### Chemical toxicity considerations.

While several chemical agents are effective in inactivating RABV under defined laboratory conditions, it is essential to recognise that many of these compounds pose significant occupational health hazards. Paraformaldehyde and formalin release formaldehyde vapours, which are irritants and sensitising agents, and are classified as human carcinogens. Their use requires adequate ventilation, appropriate respiratory protection where indicated, and strict adherence to institutional chemical safety policies. Similarly, β-propiolactone (BPL) and binary ethyleneimine (BEI), widely used for vaccine production and viral inactivation, are alkylating agents with mutagenic and potentially carcinogenic properties. These reagents should be handled only by trained personnel in controlled environments, and their use outside regulated manufacturing settings is not recommended. Oxidising agents such as sodium hypochlorite and hydrogen peroxide, while generally safer to handle, can cause chemical burns and respiratory irritation at higher concentrations, particularly when used off-label at elevated strengths or prolonged contact times. In addition, some disinfectants may lose efficacy in the presence of organic material, leading to inappropriate increases in concentration without adequate risk assessment. Accordingly, validation of inactivation protocols must consider not only virological efficacy but also operator safety, environmental ventilation, compatibility with laboratory materials, and safe waste disposal. Selection of disinfectants should be guided by a balanced assessment of antiviral activity, toxicity profile, practicality, and local resource constraints.

#### Non-chemical methods.

Non-chemical methods have also been used to inactivate RABV. A suspension of 10^9^ 50% lethal doses (LD_50_)/mL RABV was inactivated when heated at 56 °C for at least 30 min [82]. Gamma radiation of 1.2 to 1.6 megarads and ≥ 1.6 megarads inactivated RABV in street rabies-infected brains and CVS-11 rabies suspensions, respectively [83]. UV light has been used to inactivate RABV, although the reported irradiation conditions are not specified [98]. Photothermal therapy with N^+^TT-*m*CB nanoparticles, activated by near-infrared radiation (808 nm, 0.45 W cm^−2^), significantly reduced RABV titres in culture supernatants and cells after 3 min, increased the survival rate in mice by 50%, and reduced clinical manifestations [99].

## Discussion and biosafety knowledge gaps

Despite significant advancements in biosafety practices for RABV and related lyssaviruses, critical gaps remain to be addressed to enhance laboratory safety. Among these gaps, limited access to PrEP, unsafe necropsy practices, and unvalidated decontamination protocols represent the most immediate risks to laboratory personnel. A major gap remains in the availability of basic protective measures for at-risk personnel in endemic areas, which often have limited financial and technical resources. The most basic of these protective measures, PrEP, is either not provided or post-vaccination antibody titres are not routinely confirmed. This issue is further compounded by the reality that staff often perform inherently high-risk activities, such as collecting specimens for rabies exclusion, while inadequately vaccinated.

In some cases, personnel may have only partial vaccination or none at all due to vaccine shortages or lack of resources, leaving them vulnerable in hazardous working environments. Beyond vaccination, WHO guidance also emphasises the use of appropriate PPE and biological safety cabinets for aerosol-generating procedures to minimise occupational risks associated with handling rabies virus and related specimens. Additionally, there remains significant uncertainty regarding the breadth of protection provided by current rabies vaccines against non-rabies lyssaviruses. Although vaccination against the classical rabies virus provides cross-protection within phylogroup I, the level of protection against phylogroup II and divergent/unassigned lyssaviruses is poorly characterised, as in the case of IKOV [61] and WCBV [60], and remains an important area of ongoing research [11,12,14].

Improved training in sample collection and necropsy techniques is essential to minimise occupational exposure to the rabies virus during brain specimen collection. Technicians often work under high-risk conditions, and inadequate instruction in necropsy procedures can increase the likelihood of cuts, splashes, or aerosol exposure when handling suspect animals. Safer methods for obtaining diagnostic brain tissue include minimally invasive approaches, such as sampling via the occipital foramen or retro-orbital route [9,40,41], which reduce the need for extensive skull dissection while still providing suitable material for testing. Embedding these methods in standard operating procedures and providing regular hands-on training can significantly enhance biosafety and improve sample quality.

Another pressing issue concerns the effectiveness of disinfection and inactivation protocols. Although chemical and physical agents such as hydrogen peroxide, phenolic compounds, and Virkon S are known to inactivate RABV, their efficacy highly depends on specific laboratory conditions. Commonly used disinfectants for rabies-contaminated necropsy equipment did not consistently achieve complete inactivation under heavy organic load when applied according to the manufacturers' label instructions [84]. Only certain oxidising agents (e.g., sodium hypochlorite, Virkon S) and phenolic compounds achieved reliable inactivation of rabies virus, and even then only when used at higher concentrations or longer contact times than those specified in the manufacturers’ instructions (i.e., off-label use) [84]. These findings underscore that, in the presence of organic contamination, routine disinfectants may not adequately neutralise viable RABV or its RNA, and that disinfection protocols must be carefully validated to ensure both staff safety and diagnostic reliability.

Laboratory-acquired infections, although rare, highlight the need for enhanced engineering controls and rigorous safety measures. Previous incidents of exposure to aerosolised rabies virus underscore the need for additional safeguards, particularly during virus cultivation or electron microscopy procedures that may generate infectious aerosols. Importantly, while access to the pre-exposure vaccination scheme should be mandatory for any staff member who handles rabies-suspected samples, in many endemic settings, laboratory staff continue to work in rabies testing facilities without adequate vaccine protection.

## Conclusion

Addressing these biosafety gaps is essential to improve laboratory safety when handling RABV and related lyssaviruses. Priority actions include universal access to PrEP for laboratory staff, validation of inactivation protocols under realistic conditions, safer necropsy practices, and improved surveillance of laboratory incidents. Continued research, standardised protocols, and enhanced engineering controls will reduce the risk of laboratory-acquired infections, ensure a safe environment for laboratory staff and strengthen global biosafety efforts against these high-consequence zoonotic pathogens.
